# Mechanical, Thermal and Morphological Properties of Poly(lactic acid)/Epoxidized Palm Olein Blend

**DOI:** 10.3390/molecules171011729

**Published:** 2012-10-08

**Authors:** V. S. Giita Silverajah, Nor Azowa Ibrahim, Norhazlin Zainuddin, Wan Md Zin Wan Yunus, Hazimah Abu Hassan

**Affiliations:** 1 Department of Chemistry, Faculty of Science, University Putra Malaysia, UPM Serdang, Selangor 43400, Malaysia; E-Mail: hazlin@science.upm.edu.my; 2 Chemistry Department, Center for Defence Foundation Studies, National Defence University of Malaysia, 57000 Kuala Lumpur, Malaysia; E-Mail: wanmdzin@upnm.edu.my; 3 Advanced Oleochemical Technology Division, Malaysian Palm Oil Board, 43650 Bandar Baru Bangi, Malaysia; E-Mail: hazimah@mpob.gov.my

**Keywords:** biodegradable polymer, plasticizer, epoxidized palm oil, melt blending

## Abstract

Poly(lactic acid) (PLA) is known to be a useful material in substituting the conventional petroleum-based polymer used in packaging, due to its biodegradability and high mechanical strength. Despite the excellent properties of PLA, low flexibility has limited the application of this material. Thus, epoxidized palm olein (EPO) was incorporated into PLA at different loadings (1, 2, 3, 4 and 5 wt%) through the melt blending technique and the product was characterized. The addition of EPO resulted in a decrease in glass transition temperature and an increase of elongation-at-break, which indicates an increase in the PLA chain mobility. PLA/EPO blends also exhibited higher thermal stability than neat PLA. Further, the PLA/1 wt% EPO blend showed enhancement in the tensile, flexural and impact properties. This is due to improved interaction in the blend producing good compatible morphologies, which can be revealed by Scanning Electron Microscopy (SEM) analysis. Therefore, PLA can be efficiently plasticized by EPO and the feasibility of its use as flexible film for food packaging should be considered.

## 1. Introduction

The problem of accumulation of plastic waste in landfills needs immediate resolution. The impact of plastic waste on the environment includes harmful effects on wildlife, as well as on the aesthetic qualities of cities and green zones. The increasing cost of plastic waste disposal and the potential hazards from waste incineration makes synthetic plastic a waste management crisis [1]. Thus, rising environmental concerns have directed research to the development of biodegradable polymer materials as an ecologically useful alternative to plastics, primarily due to two major reasons, namely the awareness of environmental concerns and secondly the realization that our petroleum resources are finite [2].

In particular, most biodegradable polymer matrices are derived from agricultural sources, such as microbial-grown polymers (polyhydroxybutyrate, PHB; polyhydroxyalkanoates, PHAs) or those extracted from plant-origin [starch and cellulose, such as carboxymethyl cellulose (CMC)]. Some synthetic biodegradable polymers are also produced from natural monomers like poly(lactic acid) (PLA) or synthesized by chemical conversion of crude oil such as polycaprolactone (PCL) [3]. At present, PLA has been receiving much attention as the most innovative alternative to conventional petroleum-based polymers. PLA has been intensively studied due to its environmentally-friendly characteristics, biocompatibility, sustainability, as well as, potentially useful physical and mechanical properties [4].

Poly(lactic acid), a linear aliphatic polyester, are made from lactic acid monomers, ultimately made from lactose (or milk sugar) derived from renewable plant sources, such as starch and sugar. The building block of PLA is lactic acid (2-hydroxypropionic acid) which can exist as optically active D- or L-enantiomers [5]. PLA possesses high strength, good crease-retention, grease and oil resistance and excellent aroma barrier properties. Additionally, the decomposition of PLA occurs by hydrolysis, followed by biodegradation via bacteria [6].

In spite of this, PLA falls short of the properties required for potential applications in the film extrusion industry. Major limitations of PLA are due to its high brittleness, low toughness and low tensile elongation [7]. One way to improve the properties of PLA is through incorporation of plasticizers. Considerable efforts have been made to enhance the mechanical properties and flexibility of PLA by blending with plasticizers such as poly(ethylene glycol), poly(propylene glycol), citrate esters [8] and epoxidized soybean oil [9]. In response to concerns about the environment, plasticizers from natural products are currently being employed in biodegradable polymers, as a replacement for traditionally used phthalates in the plastic industry which are characterized by their high toxicity [10]. The biodegradable plasticizer employed should have functional groups such as ester and epoxy which are more likely degraded by microorganisms [11]. Epoxidized oils are a feasible option to biodegradable plasticizer and are used as raw materials in resin production. Epoxidized oil has potential as an additive for industrial applications that require properties like cheapness, good lubricity, low volatility, high index viscosity and good solvency for fluid additives [12].

Due to the sustainability of palm oil in Malaysia, new products have been developed from palm oil derivatives such as epoxidized palm olein (EPO). As Wan Rosli *et al.* [13] explain, epoxidized palm oil has been accorded priority in Malaysia having a number of commercially realizable end uses such as an effective starting material to make polyols, additives in the plastic industry, and as a pre-polymer in surface coating formulations. 

In line with this, the present study is directed towards blending PLA with EPO for the preparation of PLA/EPO blends. The melt blending method was employed as it is simpler and more cost-effective than copolymerization, and thus the more frequently used method. Subsequently, the effect of EPO loadings in PLA blend properties was investigated. Further, the properties of the PLA/EPO materials prepared were characterized through mechanical, thermal and morphological analysis.

## 2. Results and Discussion

### 2.1. Effect of EPO Loading on the Tensile Properties of Blends

**Figure 1:**
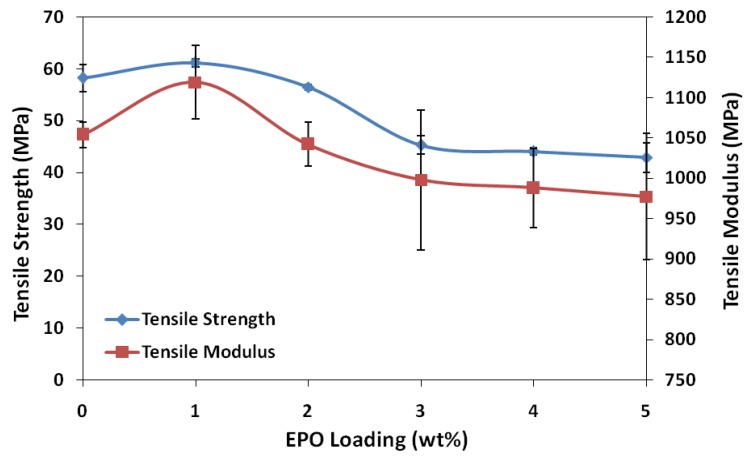
Tensile strength and modulus of neat PLA and PLA/EPO blends.

The results of tensile strength and elastic modulus of blends at different plasticizer loadings are shown in Figure 1. Addition of 1 wt% EPO into the PLA matrix produced considerable changes in the mechanical properties of the film, increasing the tensile strength and modulus of neat PLA, which is 58.2 MPa and 1,054 MPa, to 61.2 MPa and 1,119 MPa, respectively. These results may indicate a stiffer nature and higher resistance to deformation of PLA/1 wt% EPO blend compared to neat PLA. This high tensile strength and modulus is contributed by the strong interphase interaction between PLA and EPO, which reduces the stress concentration point when tensile load is applied to the blend. The higher tensile strength of PLA/1 wt% EPO blend was also due to an increase in the molecular weight (M_w_) of polymer chain, which improves the tensile strength and impact. The longer the polymer chain indicates more polymer chain entanglements are present per chain, which produces higher strength-at-break. However the changes in tensile strength and modulus of the films were less than that observed following addition of 2 wt% EPO, as demonstrated in Figure 1 [14]. 

The tensile strength decreases with increasing amounts of EPO, eventually losing about 35% of its initial strength (58.2 MPa) with 5 wt% EPO (42.9 MPa) and almost 127% (25.6 MPa) with 25 wt% EPO. When the amount of EPO is above 1 wt%, only part of the EPO locates in the interphase area, and the excess is dispersed in the PLA matrix. This affects its homogeneity and consequently reduces the tensile strength of the blends. The reduction of tensile strength above 1 wt% EPO may also be due to agglomeration which leads to poor interaction at the interphase [15]. The trend of the tensile modulus graph is similar to that of the tensile strength. Addition of EPO up to 5 wt% and 25 wt% EPO reduces the stiffness of PLA/EPO blends to almost 8% (977.2 MPa) and 24% (846.2 MPa) of the initial strength, respectively. 

**Figure 2:**
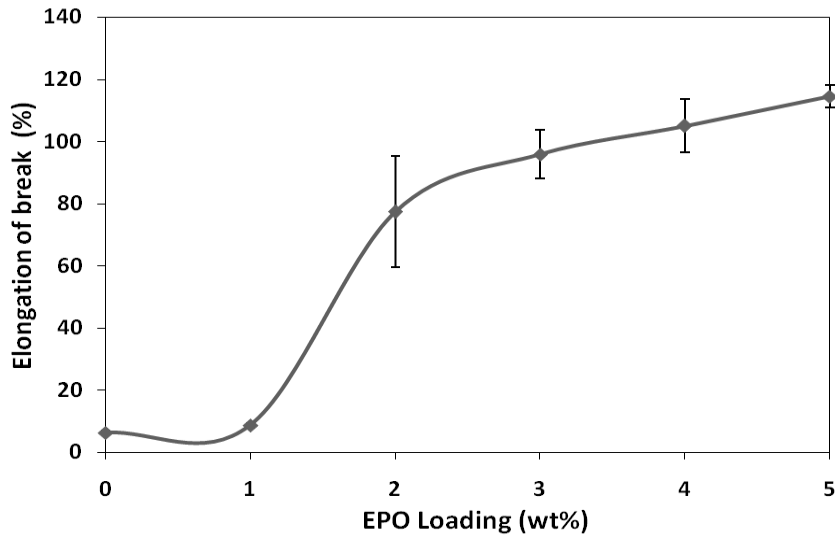
Elongation-at-break of neat PLA and PLA/EPO blends.

Figure 2 illustrates the effect of EPO on the elongation-at-break of PLA/EPO blends. The elongation at break of PLA/EPO blends with 1, 2, 3, 4 and 5 wt% EPO are 8.6%, 77.3%, 95.8%, 105.0% and 114.4%, respectively. It has been observed that elongation-at-break of blends increases significantly with increasing EPO loading. As expected, PLA blend with 5 wt% EPO loading showed a maximum elongation compared to the initial elongation of neat PLA (6.3%). This indicates that EPO reduces the intermolecular forces and increases the mobility of PLA chains, thus enhancing the flexibility of the PLA/EPO blends [16]. It has been reported that the plasticizing effect of EPO on PLA films was due to the interaction between the hydroxyl groups in PLA and epoxy groups of EPO [17].

Conclusively, the tensile test results reveal that the optimum amount of EPO loading which brings about significant improvement on the tensile strength and modulus properties is 1 wt%. Moreover, the PLA/1 wt% EPO blend appeared to be more flexible than unplasticized PLA with considerable elongation-at-break. As plasticizer functions by minimizing accumulation of intermolecular forces along polymer chains, further addition of EPO to the PLA matrix is expected to reduce the tensile strength and tensile modulus, but increase its flexibility along with elongation-at-break.

### 2.2. Effect of EPO Loading on the Flexural Properties of Blends

**Figure 3:**
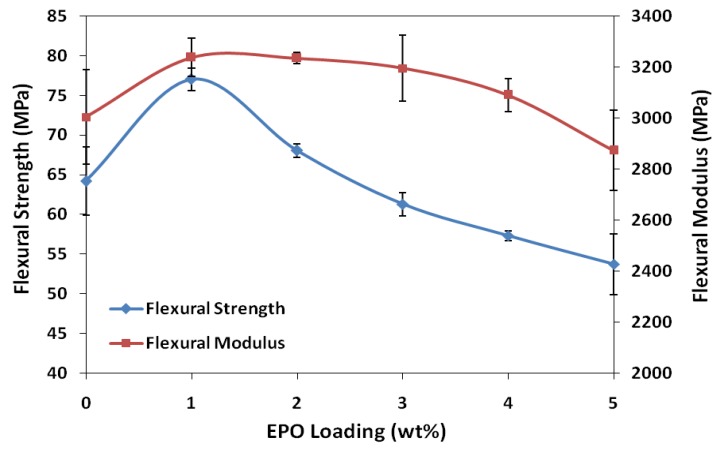
Flexural strength and modulus of neat PLA and PLA/EPO blends.

The effect of EPO on the flexural strength and modulus of PLA/EPO blends is presented in Figure 3. The flexural strength of neat PLA improved significantly upon adding 1 wt% EPO into the matrix, from 64.2 MPa to 77.0 MPa. The 20% rise indicates improved adhesion between PLA matrix and EPO, which increases the stress transfer from the polymer matrix to EPO. Similarly, the highest flexural modulus was achieved with 1 wt% EPO, with an improvement of 8% from 3,003.6 MPa (neat PLA) to 3,237.2 MPa (PLA/1 wt% EPO). Essentially, the presence of EPO in PLA/1 wt% EPO blend allows a stiffer bond formation, contributing to the enhancement in flexural properties. However, further addition of EPO decreases the flexural strength and modulus up to 43% and 13%, respectively with 5 wt% EPO loading. The drop in flexural properties was due to presence of excess EPO which reduces the stiffness of the PLA molecular chain. Moreover, increasing EPO content above 1 wt% adds to the occurrence of empty voids in the blends, which influence the local stress acting on the material [18].

### 2.3. Effect of EPO Loading on the Impact Properties of Blends

**Figure 4:**
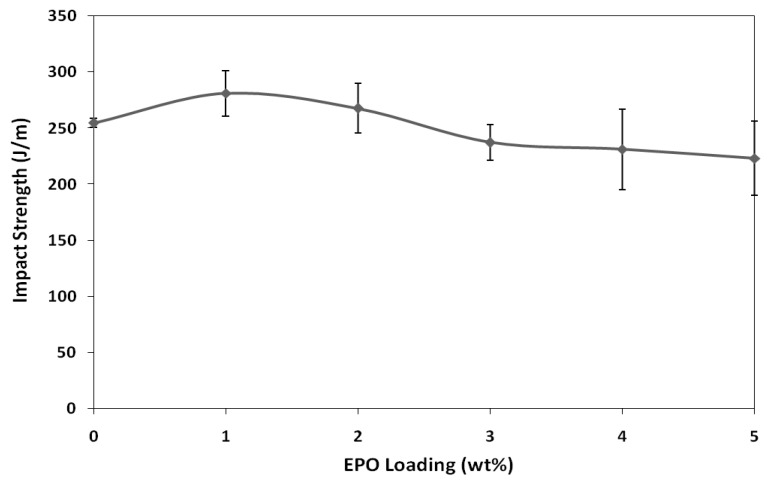
Impact strength of neat PLA and PLA/EPO blends.

The impact strength of blends are illustrated in Figure 4. The impact strength of PLA/1 wt% EPO blend is higher than neat PLA and other blend formulations. The impact strength of PLA is improved by 10% from 254.77 J/m to 281.06 J/m, with the presence of 1 wt% of EPO. This result indicates 1 wt% of EPO is sufficient to induce interactions between the PLA matrix and EPO. However, with further addition of EPO beyond the optimum amount (1 wt% EPO), the impact strength gradually decreased. Blends with higher EPO content has poor interfacial adhesion between matrix and plasticizer, causing the toughness of blends to decrease abruptly. Furthermore, the presence of excess EPO in the polymer matrix provides points of stress concentrations, hence providing sites for crack initiations. Thus, the optimum impact strength was achieved at 1 wt% of EPO [19].

### 2.4. Morphological Study of PLA/EPO Blends

**Figure 5:**
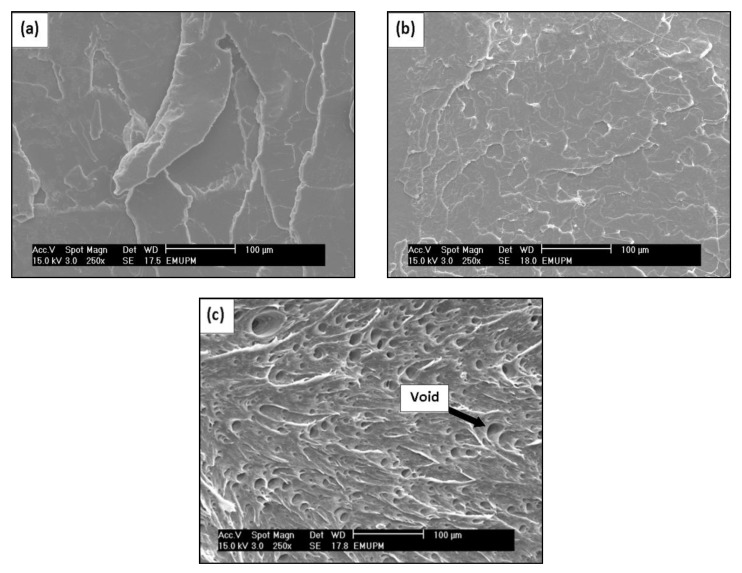
SEM images of (**a**) neat PLA, (**b**) PLA/1 wt% EPO and (**c**) PLA/5 wt% EPO blends.

The tensile fractured surface micrographs of neat PLA and PLA/EPO blends are displayed in Figure 5. It can be confirmed from Figure 5a that neat PLA undergoes a brittle failure, whereas the fractured surface of PLA/1 wt% EPO was smooth and a homogenous microstructure can be seen, indicating that no phase separation took place. No agglomerates or brittle crack behavior were observed in Figure 5b, which evidences good interfacial adhesion between the two phases of PLA matrix and EPO. This was indirectly reflected in the more efficient load transfer under stress conditions, which was apparent from the improved tensile, flexural and impact properties of the blend. Moreover, this observation is consistent with the DMA results [15,20].

When the EPO content was increased to more than 1 wt%, the dispersion of plasticizer in the matrix became inhomogenous, reflecting in a decrease of tensile and impact properties. Conversely, the marked increase in the break elongation with increasing EPO content was due to the reduced stiffness associated with high concentrations of plasticizer. Figure 5c revealed poor interfacial adhesion properties and the presence of empty voids caused by debonding, when EPO was present in excess [21]. Overall, addition of 1 wt% EPO to PLA matrix produces single phase morphology and improves the properties of PLA.

### 2.5. Fourier Transform Infrared (FTIR) Analysis of PLA/EPO Blends

**Figure 6:**
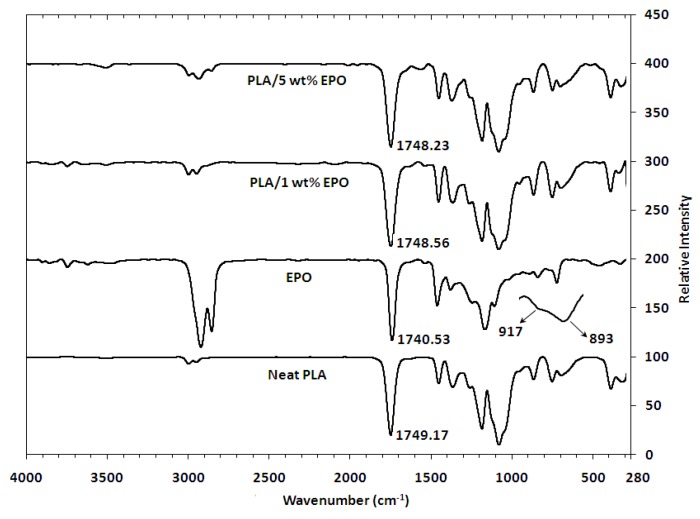
FTIR spectrum of neat PLA, EPO and PLA/EPO blends.

FTIR spectroscopy is a technique that is sensitive to intermolecular interactions. In this study FTIR spectroscopy was used to monitor the absorption peak shifts in specific regions to determine the known functional group interactions of the PLA with EPO. The FTIR spectrum of neat PLA, EPO and PLA/EPO blends are depicted in Figure 6. The spectrum shows C=O stretching overtones at 3660–3400 cm^−1^, CH_3_ stretching at 3000–2850 cm^−1^ and O–C=O stretching at 1190–1080 cm^−1^. An important characteristic of ester bonds is C=O stretching at 1750–1745 cm^−1^ for PLA, and 1743–1740 cm^−1^ for EPO. Additionally, the EPO spectrum displayed small intensity signals at 950–850 cm^−1^ and around 1250 cm^−1^ indicative of C–O–C stretching from oxirane vibrations. The signal at 1250 cm^−1^ usually overlay with others, mainly C–O ester which is present in oils. The diminishing of these bands is complementary evidence that the desired reaction is taking place [22].

**Figure 7:**
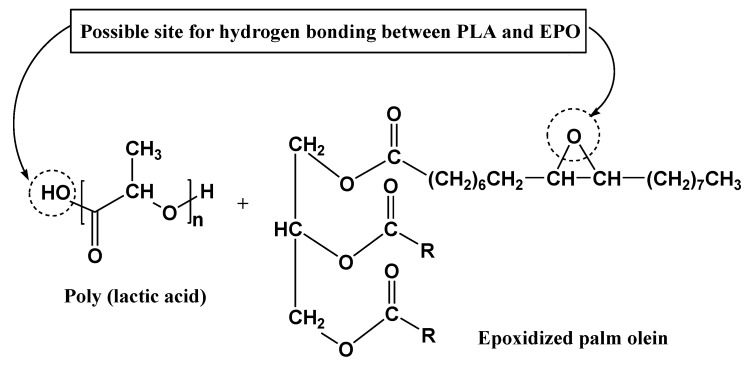
Proposed chemical interactions between PLA and EPO.

As the optimum loading of EPO is small, 1 wt%, it exhibited an insignificant shift in the infrared spectrum compared to neat PLA. Therefore, the infrared spectra of PLA/1 wt% EPO blends were found to be similar to that of neat PLA. However, there were minute differences which could be taken into account as indicating that interactions most likely took place between PLA and EPO. Since the amount of EPO (1% with respect to PLA) present in the blend is small, the C=O stretching of EPO peak might be overlapping with the carbonyl band of PLA. However, a small shift from 1749.17 cm^−1^ (neat PLA) to 1748.56 cm^−1^ (PLA/1 wt% EPO) was observed. This downshift in the absorption peak indicates the miscibility and interaction of PLA and EPO [23]. The shift may possibly due to an interaction between the hydroxyl group of PLA and epoxy group of EPO through hydrogen bonding (H-bonding) interaction, resulting in enhanced morphological properties which can be verified through SEM analysis. A proposed possible site for interaction between PLA and EPO is shown in Figure 7 [17,24].

Furthermore, with 5 wt% EPO the C=O shift was seen at 1748.23 cm^−1^, indicating a C=O frequency shift to lower wavenumbers with increasing EPO content. The peak intensity of free O-H vibrations at 3800–3500 cm^−1^ and C-H vibrations at 3000–2800 cm^−1^ increased gradually with EPO content which reveals the effect of adding EPO. However, a small amount of hydroxyl group (O–H) in the blend could be attributed to the possible terminal hydroxyl groups in the PLA main chain which was released during the interaction between PLA and EPO [17]. Finally the presence of a new weak absorption band around 1600–1500 cm^−1^, which appears with excess EPO, corresponds to the stretching vibration of the COO– group.

### 2.6. Dynamic Mechanical Analysis of PLA/EPO Blends

**Figure 8:**
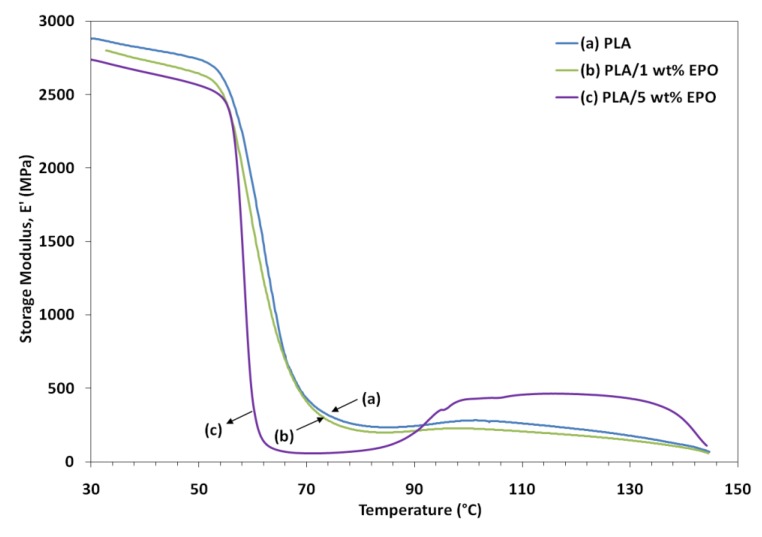
Storage modulus of neat PLA and PLA/EPO blends as a function of temperature.

**Figure 9:**
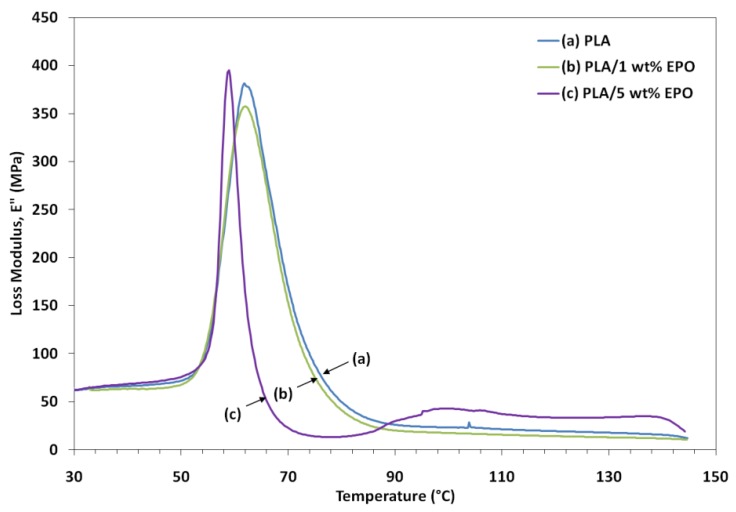
Loss modulus of neat PLA and PLA/EPO blends as a function of temperature.

**Figure 10:**
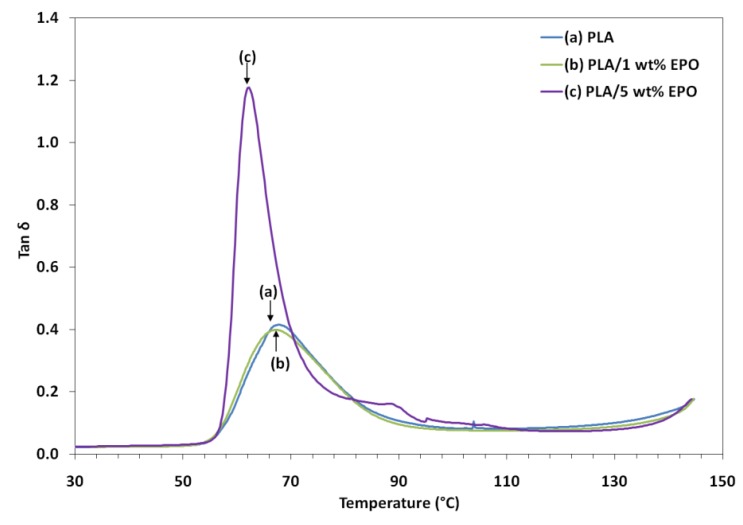
Loss factor of neat PLA and PLA/EPO blends as a function of temperature.

Variations of dynamic storage modulus (E'), loss modulus (E") and tan δ of the samples as a function of temperature are shown in Figure 8, Figure 9 and Figure 10, respectively. The lower E' of the PLA/EPO blends compared to neat PLA can be seen in Figure 8, indicating an increase in the flexibility of PLA incorporated by the EPO. In addition, the E' of the blends decreased with an increased in the EPO content below 80 °C. There was also a large drop in E' around 50–80 °C corresponding to the glass transition region, and then it started to rise around 90 °C. The modulus drop associated with the T_g_ of plasticized PLA decreases almost linearly with increasing plasticizer concentration, from 67.9 °C for neat PLA to 67.3 °C and 62.2 °C for 1 and 5 wt% EPO loading, respectively. The rise in E' was due to crystallization on heating (cold crystallization) [9].

Loss modulus represents the melt viscosity of a polymer, also known as flow of matter. A decrease in the peak intensity of loss modulus is observed for PLA/1 wt% EPO blend which signifies a decrease in the melt viscosity of the corresponding blend. Addition of 1 wt% EPO reduces the viscosity of the blend through the replacement of polymer-polymer hydrogen bonding by polymer-plasticizer hydrogen bonding. This improves the processability and workability of PLA by reducing viscosity at processing temperatures. Besides, the fall in the modulus is also attributed to an energy dissipation phenomenon involving cooperative motions of the polymer chains. When 5 wt% EPO was incorporated into the PLA matrix, the peak intensity shifts to a lower processing temperature and a slight increase in the loss modulus was observed. This indicates that higher EPO loading may considerably increase the viscosity of polymer compositions by acting as a good solvent or plasticizer [25].

Tan δ can be defined as the damping term that can be related to the impact resistance of a material. As the damping peak occurs in the region of T_g_ (where the transition of material from a rigid to an elastic state takes place), it is associated with the movement of small groups and chains of molecules within the polymer structure. Figure 10 also shows that T_g_ determined from the tan δ peak were shifted to a lower temperature with increasing EPO content in the blends. It is observed that the PLA/5 wt% EPO showed a higher tan δ peak (≈1.2) as compared to PLA/1 wt% EPO (<0.4) and neat PLA (>0.4). This indicates that the higher damping affects at the interfaces, leading to poorer interface adhesion in PLA/EPO (95/5).

PLA/EPO (95/5) blend with poor interface bonding tends to dissipate more energy than that with good interface bonding, PLA/EPO (99/1). With additional EPO content, the strain is applied to the blend to a higher degree, leading to more dissipative component of the blend. The width of the tan δ peak also becomes narrower than that of the pure matrix system with increasing addition of EPO. This behavior suggests that there are lesser molecular relaxations in the blend compared to those present in the pure matrix. In addition, the width of the tan delta peak is indicative of the decreased volume of the interface with further addition of EPO. This unable it to absorb energy at fracture when exposed to sudden impact and results in lower impact strength [26].

As seen in the graph, incorporation of 1 wt% EPO reduces the tan δ peak height of PLA by restricting the movement of the PLA molecules, which signifies improvement in the interfacial bonding of the blends. Besides, the slightly broader width of the tan delta peak indicates an increase in the molecular relaxation, enable the blend to absorb more energy at fracture, leading to higher impact strength. Moreover, the introduction of EPO reduces the T_g_ of the tan δ peak to a lower temperature. The shift in T_g_ can be associated with the increased mobility of the chains by the addition of plasticizer. These results are consistent with the elongation of break from tensile test and the DSC results.

### 2.7. Thermogravimetric Study of PLA/EPO Blends

**Figure 11:**
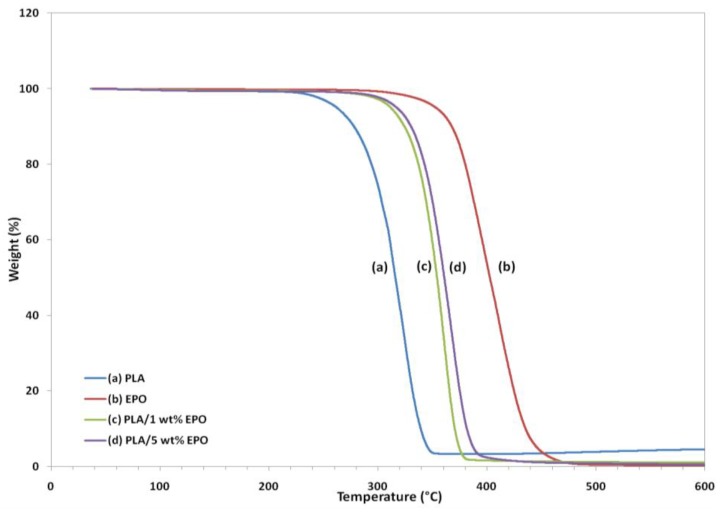
TG thermograms of neat PLA and PLA/EPO blends.

**Figure 12:**
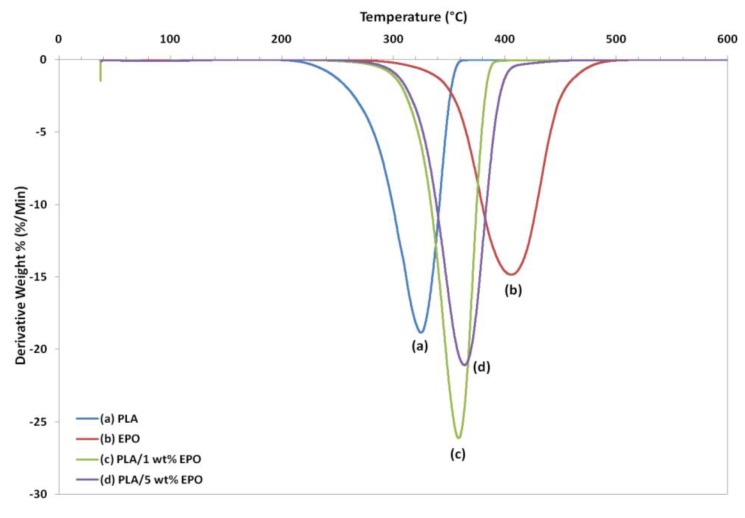
DTG thermograms of neat PLA and PLA/EPO blends.

Thermogravimetric analysis (TGA) gives information on the weight loss due to degradation as a function of temperature. Figure 11 and Figure 12 represent the weight loss curves (TG) and the corresponding derivative (DTG), respectively. For comparison purposes, the TGA profile of the pure compounds are also included in the plot. The characteristic thermal parameters selected were onset temperature, which is the initial weight loss temperature, and maximum degradation temperature (T_max_), which is the highest thermal degradation rate temperature.

Thermal degradation of neat PLA, EPO and PLA/EPO blends takes place in a single weight loss step, which can be evidence from the DTG curves. The profile for the pure compounds showed that the PLA has lower thermal stability than EPO, because its degradation peak was at 324.8 °C and it was completely decomposed at 363.1 °C, while EPO showed peak degradation at 405.1 °C and was fully degraded at 504.3 °C. The onset degradation temperatures of PLA/EPO blends are higher than that of neat PLA. Neat PLA has an onset temperature of 211.9 °C, which is increased to 272.3 °C and 270.4 °C when 1 wt% and 5 wt% EPO respectively incorporated into the blends. The results show that the initial degradation temperature is significantly enhanced by 50 °C and T_max_ greater than 30 °C, with approximately 99% weight loss when EPO was added into the PLA matrix.

The improved thermal stability is attributed to the presence of EPO (onset temperature = 282.9 °C) dispersed in the polymer matrix. This reassembling creates a protective physical barrier on the surface of the material which hinders the permeability of volatile degradation products out from the blend and eventually helps delay the degradation of the blend. However, blend with 1 wt% EPO showed higher thermal stability compared to the 5 wt% EPO, indicating better interaction in the PLA/1 wt% EPO blend [27].

The T_max_ increases significantly, while onset thermal degradation temperature decreases insignificantly when EPO content was raised from 1 to 5 wt%. It can be observed that the T_max_ of PLA/5 wt% EPO is higher by 4 °C than 1 wt% EPO blend. This is ascribed to the presence of excess EPO in the matrix. Besides that, at 350 °C PLA/5 wt% EPO blend exhibited much more weight loss (82%) than PLA/1 wt% EPO blend (70%) despite the similar weight loss at 300 °C. This is possibly related to the large difference between the T_g_ temperatures of the two starting material which adds to the degradation mechanism [28].

### 2.8. Differential Scanning Calorimetry Study of PLA/EPO Blends

**Figure 13:**
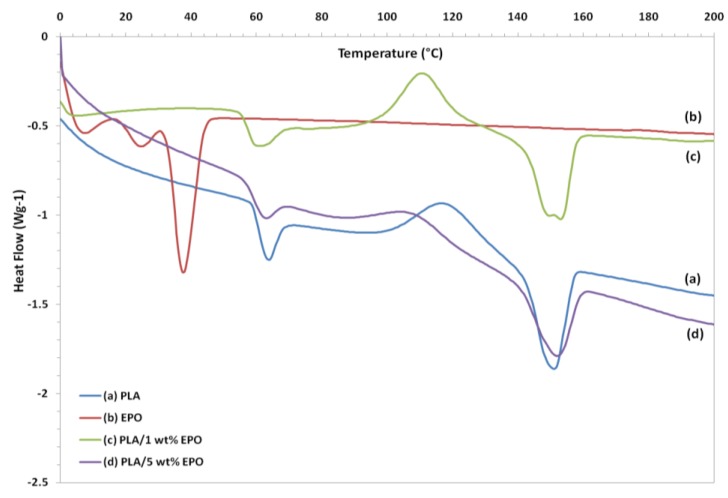
DSC thermograms of neat PLA and PLA/EPO blends.

The DSC heating thermograms of neat PLA, EPO and plasticized PLA films are summarized in Figure 13. The curves of plasticized PLA exhibited three thermal transitions, *i.e.*, glass transition (T_g_), crystallization (T_c_), and melting (T_m_) temperatures [29]. Neat PLA showed a T_g_, T_c_ and T_m_ peak located at 63.55 °C, 117.89 °C and 151.10 °C, respectively. The plasticizer decreased the glass transition temperature (T_g_) of neat PLA from 63.55 °C to 59.90 °C and 56.76 °C by adding 1 and 5 wt% of EPO, respectively. The decrease in T_g_ was enhanced with a higher EPO content due to plasticizing effect and indicates that EPO is miscible with PLA. Addition of EPO enhanced PLA chains mobility which made it easier for them to fold into crystalline lattice [30]. This result is similar to that described for PLA plasticized with epoxidized soy bean oil reported by Fathilah *et al*. [9].

Furthermore, the crystallization temperature (T_c_) decreases with the addition of EPO. Addition of 1 wt% EPO decreased the T_c_ to 110.79 °C, while for 5 wt% plasticized PLA film the T_c_ was further decreased to 105.28 °C. It can be observed that, as the content of EPO increased to 5 wt%, the T_c_ peak became broader and nearly indistinguishable. The T_c_ of PLA gradually decreased by the addition of EPO, which suggests that EPO enhances the ability to cold crystallization of PLA. It is evident that the T_c_ of PLA decreases in parallel with the shift in T_g_. Such a decrease in T_g_ and an enhancement in crystallization process are commonly observed for plasticized PLA systems and are due to the increase in segmental mobility of the PLA chains by plasticization [9].

The DSC thermogram of EPO exhibits two separate melting temperature peaks (T_m_) at 24.46 °C and 37.69 °C. As observed from Figure 13, the DSC thermogram of PLA/1 wt% EPO exhibits two distinct peaks of melting temperature at 149.8 °C and 153.1 °C, respectively. According to Hala *et al.* [31], the lower melting endotherm corresponds to the crystalline phase of PLA/EPO soft segments and the higher one to the crystalline phase of hard segments. Conversely, in the heating scan of PLA/5 wt% EPO only one T_m_ peak appeared at 152.4 °C resembling to the crystalline phase of soft segments. In addition, the upward shift in T_m_ of this blended film (PLA/5 wt% EPO) indicates the decreased miscibility of the components in the blends [23].

The crystallinity of PLA/EPO blends was estimated according to the enthalpy obtained from the DSC curves. The percentage of crystallinity, X_c_ were calculated using the following equation:

$$X_{c}(\%)=\frac{\left|\Delta H_{m}-\Delta H_{c}\right|}{\left|\Delta H_{m}(100\%)\right|}\times 100\%$$

Here, ΔH_c_ is the enthalpy of crystallization (J/g), ΔH_m_ is the enthalpy of dissolution (J/g), and ΔH_m_ (100%) is the enthalpy of dissolution (93 J/g) of PLA with a crystallinity of 100% [32]. In this investigation, the neat PLA and PLA/1 wt% EPO showed X_c_ of approximately zero, which means they are amorphous. The X_c_ increased by blending 5 wt% EPO to 10.3%, indicating it is semi-crystalline. Therefore, the degree of crystallinity increases when the EPO content is increased.

### 2.9. X-ray Diffraction (XRD) Study of PLA/EPO Blends

**Figure 14:**
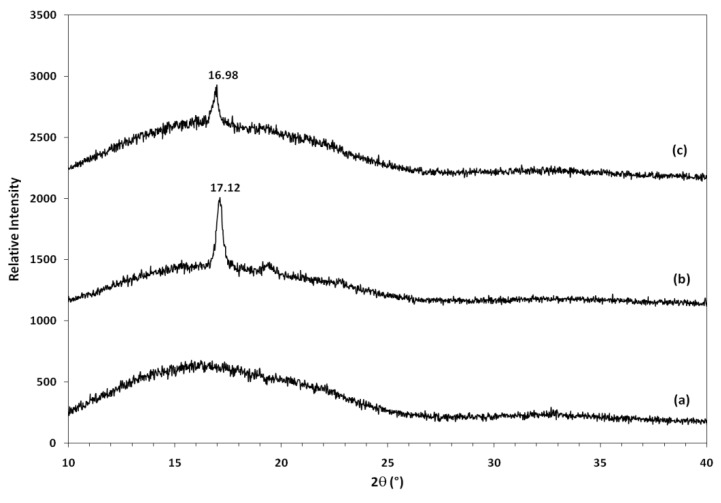
XRD of (**a**) neat PLA, (**b**) PLA/1 wt% EPO and (**c**) PLA/5 wt% EPO blends.

The X-ray diffraction (XRD) patterns of plasticized PLA blends are compared in Figure 14. In addition to the broad diffraction peak centered at 16.22°, which can be assigned to neat PLA, a crystalline peak was observed at the 2θ of 17.12° and 16.98° for PLA/EPO blend with 1 and 5 wt% EPO, respectively. XRD results illustrated that PLA/EPO blends are semi-crystalline. These facts confirmed that the introduction of the EPO component into PLA chain has altered the regularity of the polymer, but the extent of their effects may be connected with the amount of EPO components present in the blend [33].

The corresponding basal spacing of neat PLA, PLA/1 wt% EPO and PLA/5 wt% EPO blends are 5.47, 5.18 and 5.22 Å, respectively. The intensity of the characteristic peak of PLA/5 wt% EPO was reduced significantly compared to PLA/1 wt% EPO blend, but almost retains its position, indicating the formation of a conventional or phase separated blends. Such structure was expected since EPO was present in excess, increasing the interplaner spacing compared with that of PLA/1 wt% EPO blend, which would shift the peak towards a lower angle. However, the existence of sharp peaks showed that the blends still retain an ordered structure [34].

EPO favours crystallization for the PLA, because hydrogen bonds facilitate the alignment of the polymer chains, yielding higher crystallinity. Increasing EPO content causes formation of an excess number of hydrogen bond linkages and results in a more complex structure and therefore the alignment of the polymer chains is disturbed, forming a semi-crystalline structure [33]. The absence of a crystalline peak in neat PLA indicates that it is amorphous, followed by PLA/5 wt% EPO and PLA/1 wt% EPO blend having the highest crystallinity. 

The XRD results of neat PLA and PLA/5 wt% EPO blend verify the differential scanning calorimetry (DSC) crystallinity findings. However, the XRD result of PLA/1 wt% EPO blend contradicts the percentage of crystallinity (X_c_) calculated via DSC. Crystallinity calculated from DSC results revealed that PLA/1 wt% EPO is amorphous instead of semi-crystalline as obtained from XRD. This was due to the different conditions of the instrumental analysis techniques (DSC and XRD) and the distribution of amorphous and crystalline region in the semi-crystalline blends, which affects the crystallinity data obtained. The percentage of crystallinity calculated from DSC is temperature dependent, based on enthalpy of crystallization and fusion. As these values are obtained at higher temperatures, this result cannot be compared with XRD analysis measured at room temperature. This may cause the crystallinity data obtained from DSC and XRD to contradict each other.

## 3. Experimental

### 3.1. Materials

Poly(lactic acid) pellets, commercial grade 4042D with 1.24 g/cm^3^ density, were purchased from NatureWorks^TM^ LLC (Blair, NE, USA). The polymer was dried for at least 3 h at 50 °C before being used in the blend preparation. Epoxidized palm olein (EPO, epoxide content = 3.2%) was supplied by Advanced Oleochemical Technology Division (AOTD), Malaysian Palm Oil Board (MPOB).

### 3.2. Preparation of PLA/EPO Blends

The PLA/EPO blends were produced by the melt blending technique using a twin counter-rotating mixer (Brabender internal mixer, MELCHERS, Duisburg, Germany) at 170 °C for 15 min and rotor speed of 50 rpm. Varying amounts of EPO (1, 2, 3, 4, and 5, in wt%) were then mixed together with PLA pellets in a mixing chamber. After melt blending, the sheets were prepared using a hydraulic hot-press (Hsin-Chi Machinery Company Ltd., Chiai, Taiwan) with a pressure of 110 kg/cm^3^ at 160 °C for 10 min, and then, cooled to room temperature to produce sheets of uniform 1 mm and 3 mm thickness. 

### 3.3. Tensile Test

Tensile testing was carried out using an Instron Universal Testing Machine (Model 4302 Series IX) based on ASTM D638 (Type V). Seven dumbbell shape specimens were prepared from each composition. The average thickness and average width of the gauge section of each specimen were calculated. The test was conducted at a constant crosshead speed of 5 mm/min, load cell of 1 kN and a gauge length of 10 mm. Tensile strength, tensile modulus and elongation at break were obtained using Instron Series IX software. 

### 3.4. Flexural Test 

Flexural test was conducted in accordance with ASTM D790, using an Instron Universal Testing Machine (Model 4302 Series IX) equipped with a 1 kN load cell. Seven specimens in rectangular shape with the dimension 127 mm × 12.7 mm × 3 mm of size were tested for each composition. Flexural strength and flexural modulus were obtained at constant crosshead speed of 3 mm/min. 

### 3.5. Izod Impact Test

Impact test was conducted using an Izod Impact Tester (International Equipments, Mumbai, India) with a 453 g (1.0 pound) pendulum according to ASTM D256. Five specimens with length 63.5 mm, 12.7 mm width and thickness approximately 3.0 mm, were tested for each composition. The impact strength (J/m) was calculated by dividing the energy obtained (J) with the thickness of specimen (m). 

### 3.6. Scanning Electron Microscopy (SEM)

Morphology analysis of tensile fractures surface of blends was observed by Scanning Electron Microscopy (Model Philips XL 30), with an acceleration voltage of 20 kV. In order to avoid electrostatic charging during electron irradiation, the specimens were sputter coated with gold a few nanometres in thickness, in vacuum conditions prior to each examination.

### 3.7. Fourier Transform Infrared (FTIR) Analysis

FTIR spectra of blends were recorded using a Fourier Transform Infrared (FTIR) Spectrometer (Perkin-Elmer: Model 1000 Series) instrument equipped with a universal attenuated total reflectance (UATR) accessory. The spectra were recorded between 4,000 cm^−1^ and 280 cm^−1^ frequency ranges, with 4 cm^−1^ spectral resolution. The data were analyzed using the program FTIR Spectrum Software (Perkin Elmer).

### 3.8. Dynamic Mechanical Analysis (DMA)

Thermal dynamic analysis (DMA) was performed according to ASTM D5023 on a dynamic mechanical analyzer (Perkin-Elmer PYRIS Diamond DMA), using bending mode. The temperature scan was from ambient temperature (25 °C) to 100 °C at a constant heating rate of 2 °C/min and the frequency of dynamic force of 1 Hz, under nitrogen atmosphere. The storage modulus (E'), loss modulus (E''), and loss factor (tan δ) of each specimen were obtained as a function of temperature.

### 3.9. Thermogravimetric Analysis (TGA)

The thermal stability of the samples was studied by using the Perkin Elmer TGA7 Thermogravimetric Analyzer. The weight of the samples used was approximately 10.0 mg and were heated from 25 °C to 500 °C at the heating rate of 10 °C/min. The analysis was carried out in nitrogen atmosphere with nitrogen flow rate of 20 mL/min. The weight loss of samples were recorded and plotted as a function of temperature.

### 3.10. Differential Scanning Calorimetry (DSC) 

The melting and crystallization behavior testing of the blends was performed using a DSC analyzer machine (Model DSC822, Mettler Toledo, Columbus, OH, USA). The scan was carried out at the rate of 10 °C/min from 0 °C to 200 °C with a flow rate of nitrogen gas of 50 mL/min. The method utilizes differential heat flow and temperature, normally associated with transition in materials. 

### 3.11. X-ray Diffraction (XRD) Analysis

X-ray diffraction measurement was carried out by using a Shimadzu XRD 600 X-ray diffractometer with CuKα radiation (λ = 1.542 Å) operated at 30 kV and 30 mA. Data were collected within the range of scattering angles (2θ) of 10° to 40° at the rate of 2 °/min. The basal spacing was derived from the peak position (*d*_001_ reflection) in the XRD diffractogram according to the Bragg equation (λ = 2*d* sinθ).

## 4. Conclusions 

In the present work, the mechanical, thermal, and morphological properties of PLA/EPO blends at different EPO loadings have been investigated. The optimum EPO loading for enhanced mechanical and thermal properties of PLA/EPO is 1 wt%. SEM results evidence good interfacial adhesion between the two phases of the PLA matrix and EPO. This is indirectly reflected in improved tensile, flexural and impact properties of the blend by 5%, 6% and 10%, respectively. Moreover, DMA measurements indicate that the plasticization with 1 wt% EPO leads to a decrease in storage modulus, due to an improved flexibility of the PLA. From the DSC results, a shift to lower glass transition temperature was observed due to an increase in the mobility of polymer chains by the addition of EPO. This composition also displayed lower crystallinity, indicating it is amorphous. In addition, TGA thermograms revealed a substantial increase in the matrix degradation temperatures and thermal stability by 28.5%. Considering all the analyses performed, it can be concluded that these blends enhanced the overall properties of neat PLA. This makes PLA/1 wt% EPO blend an interesting multifunctional material for possible industrial applications.
